# Supplementary material for: Brooklyn plots to identify co-expression dysregulation in single cell sequencing
Source: NAR Genom Bioinform. 2024 Jan 11;6(1):lqad112. doi: 10.1093/nargab/lqad112 (PMC10782911; doi:10.1093/nargab/lqad112)
Supplement: lqad112_Supplemental_Files [file lqad112_supplemental_files.zip › Supplementary Files.pdf]

Brooklyn plots to identify co-expression dysregulation in single cell sequencing

Arun H. Patil[1] , Matthew N. McCall[2,3] and Marc K Halushka[4*]

1 Lieber Institute for Brain Development, Baltimore MD, USA

2 Department of Biostatistics and Computational Biology, University of Rochester Medical Center, Rochester, NY, USA.

3 Department of Biomedical Genetics, University of Rochester Medical Center, Rochester, NY, USA.

4 Pathology and Laboratory Medicine Institute, Cleveland Clinic Foundation, Cleveland OH, USA

Supplementary Materials

Supplementary Figure 1

Supplementary Tables 1-3

Supplementary Methods

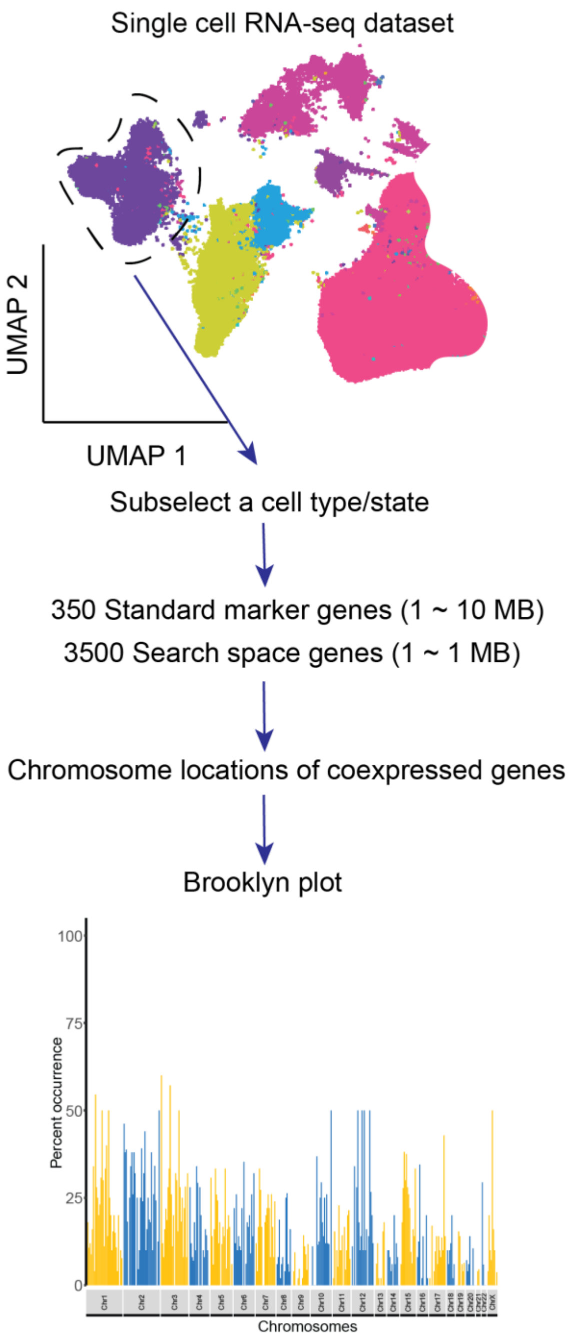

Supplementary Figure S1. Schematic of the Brooklyn plot method. From a h5ad file, a single cell type/state is subselected from a scRNA-seq or snRNA-seq dataset (dotted circle in the UMAP plot). Standard marker genes and a search space are established. Co-expression by chromosome is established and a Brooklyn plot is generated.

Supplementary Table S1. The percent of genes across each chromosome. Data from Ensembl.

Chromosome	Protein Coding Genes	% To All Genes
1	2061	10.08%
2	1299	6.35%
3	1081	5.29%
4	757	3.70%
5	882	4.31%
6	1051	5.14%
7	1010	4.94%
8	701	3.43%
9	778	3.80%
10	730	3.57%
11	1317	6.44%
12	1037	5.07%
13	322	1.57%
14	821	4.01%
15	617	3.02%
16	863	4.22%
17	1186	5.80%
18	266	1.30%
19	1476	7.22%
20	546	2.67%
21	221	1.08%
22	495	2.42%
X	859	4.20%
Y	63	0.31%
mtDNA	13	0.06%
All genes	20452	
Avg across all chromosomes		4.35%

Supplementary Table S2. Metadata on 43 samples used in this study.

See Excel File

Supplementary Table S3. Various modifications to the Brooklyn plot method were used to explore the final percent co-expression values. A) Comparison of the number of co-expressed genes to each standard gene to evaluate for diseased cardiomyocytes and endothelial cells using a Pearson correlation. B) Modifications of the total number of highly expressed genes, the number of standard genes and the number of co-expressed genes by Kendall's tau. C) Evaluation of a Bayesian correlation of the same cells, using the standard 3500, 350 and 50 approach. As can be seen, the method is robust to the number of standard markers (200-500) and total number of highly expressed genes. The highest correlation values were identified with the fewest correlated genes (25), indicating a ~12.5 MB search space to each side of the marker gene. As the search space increased, the number of co-expressed genes on the same chromosome decreased noticeably for both cell types with and without elevated Brooklyn plot co-expression values.

See Excel File

Supplementary Methods

The Brooklyn plot package is available at GitHub (https://github.com/arunhpatil/brooklyn). It can be installed using conda "conda install -c bioconda brooklyn_plot" or PyPi ("python3.8 -m pip install --user brooklyn_plot")[1]. The input is an h5ad file of a single cell/nucleus sequencing dataset obtained from any source. If the single cell dataset of interest is not in h5ad format, it can be converted through sceasy (https://github.com/cellgeni/sceasy)[2].

To demonstrate the usefulness of the Brooklyn plot, we provide the h5ad file "Cardiomyocytes" from Cellxgene (https://cellxgene.cziscience.com/)[3]. The dataset is loaded with the Scanpy package and is filtered to the user's interests. In this example, only cardiomyocytes from left ventricles of patients with *TTN* mutant dilated cardiomyopathy were subselected. Other filters can be chosen with the goal to settle on a single cell type population. For these subsetted cells, raw gene expression abundance is ranked and the top 3500 expressed genes becomes the full search space, discarding more lowly expressed genes. Gene chromosomal locations are obtained from Biomart annotations. These genes are sorted by chromosome location and every 10th gene is used as a standard for ~10 MB coverage of the entire genome (n=350 genes). The detailed analysis steps are documented as a Jupyter notebook (https://brooklyn-plot.readthedocs.io/en/latest/notebooks/example_TTN_CV_DCM.html) along with a link to the test dataset. . Output files are a subsetted h5ad file, the Biomart annotations, a list of 350 standard (to be queried) genes, and the 3500 genes in the search space.

Other parameters were evaluated with more or fewer genes. We observed no improvement in the method by increasing (or decreasing) the search space of genes (3500) or the number of standard genes (350). However, the software is flexible to allow a user to explore these parameters to perhaps identify changes in the behavior of the method.

These files are provided as input to the “Brooklyn_plot” package where an internal module “brooklyn_arch()” converts the h5ad to a Numpy array and an iterative Pearson correlation for each query gene across the search space is performed using the “stats.pearsonr” method of Scipy. The Brooklyn package offers a Kendall’s tau (a SciPy method) and a Bayesian correlation (BaCo) method implemented in R[4]. This generates a folder of CSV files for each gene that contains correlation coefficient (*r*), P-value and Bonferroni corrected P-value, $-\log_{10}$(P-value) and gene coordinates for all correlated genes above a Bonferroni corrected P-value of 0.05. The “summarize()” function in the Brooklyn plot package, determines the percent of the top 50 co-expressed genes (to all 350 standard genes) that localize to the same chromosome as the queried standard gene. Subsequently, this resultant summary file is represented as a Brooklyn plot in PDF format using the “ggplot2” package in R. The Brooklyn plot package utilizes “concurrent.futures” functionality and offers parallel processing across multiple threads for productivity gains.

Functional limitations of the data used for Brooklyn plots

Based on empirical data, subselected cell types with <250 cells may perform poorly due to limited data. Datasets that have curated down to a reduced number of total genes (<300), do not work well. Subselected cell types that cluster poorly on dimensionality reduction plots also behave poorly by this method.

Combined Brooklyn plots

For the 15-cell type scRNA-seq and 7-cell type snRNA-seq samples, the individual standard genes varied between samples. To overcome this, data was merged based on chromosome location in 1 MB intervals across the genome. Then all 1 MB intervals with a denominator of <50 genes were removed and the remaining values (percents of co-expressed genes from the same

chromosome) were plotted. For the 15-cell type figure, this was 1065 measures. For the 7-cell type figure, this was 1083 measures.

Localization of peak co-expression

Three standard genes (*NDUFS1, REV3L, DNAJC1*) were selected from different chromosome locations from the *TTN* DCM output files. They were chosen for each having 98+% of the top 50 co-expressed genes being on the same chromosome. The location of the 49 or 50 co-expressed genes were solved relative to all genes on the chromosome (within the 3500 gene search space). A moving sum of the co-expressed genes for 15 adjacent genes was determined across the chromosome. Values ranged from 0 to 13. Then a moving average for 30 adjacent counts was used to smooth the values for plotting, which was performed in R.

Additional References

1 Gruning, B. *et al.* Bioconda: sustainable and comprehensive software distribution for the life sciences. *Nature methods* **15**, 475-476, doi:10.1038/s41592-018-0046-7 (2018).

2 Cakir, B. *et al.* Comparison of visualization tools for single-cell RNAseq data. *NAR Genom Bioinform* **2**, lqaa052, doi:10.1093/nargab/lqaa052 (2020).

3 Reichart, D. *et al.* Pathogenic variants damage cell composition and single cell transcription in cardiomyopathies. *Science* **377**, eabo1984, doi:10.1126/science.abo1984 (2022).

4. Sanchez-Taltavull, D. *et al.* Bayesian correlation is a robust gene similarity measure for single-cell RNA-seq data. *NAR Genom Bioinform*, **2**, lqaa002 (2020).
